# Quantifying the effects of repeated dyeing: Morphological, mechanical, and chemical changes in human hair fibers

**DOI:** 10.1016/j.heliyon.2024.e37871

**Published:** 2024-09-12

**Authors:** Sangwoo Kwon, Seoyoon Lee, Jihui Jang, Jun Bae Lee, Kyung Sook Kim

**Affiliations:** a Department of Biomedical Engineering, College of Medicine, Kyung Hee University, Seoul, 02447, Republic of Korea; b Department of innovation, Innovation Lab, Cosmax R&I, Gyeonggi-do, Republic of Korea; c Department of Biomedical Engineering, Graduate school, Kyung Hee University, Seoul, 02447, Republic of Korea

**Keywords:** Hair dyeing, Biomechanical property, Stiffness, Atomic force microscopy, FT-IR

## Abstract

As hair dyeing gains popularity across all age groups, concerns about the potential damage caused by chemical treatments are also on the rise. Chemical dyes have a multifaceted impact on hair fibers, affecting their morphology, physical structure, and protein composition. In a comprehensive study, we investigated the alterations in morphological and mechanical properties, as well as the chemical composition of hair fibers following continuous dyeing. Our analysis employed various techniques, including atomic force microscopy (AFM), Fourier transform infrared (FT-IR) spectroscopy, and tensile strength measurements. To assess the cumulative damage resulting from repeated dyeing, we progressively increased the number of dyeing up to 10. Surprisingly, even a single dyeing session inflicted noticeable harm on the hair. However, the detrimental effects escalated significantly when hair underwent three or more consecutive dye treatments. While the mechanical properties and protein composition exhibited non-linear changes with increasing the number of dyeing, we observed that nanoscale damage to the cuticle surface intensified proportionally with the number of dyeing. These results highlight the critical need to consider the impacts of hair dyeing practices on both the health and the structural integrity of hair.

## 1 Introduction

Hair is an important part of the body, serving both aesthetic and functional purposes. It contributes to personal expression, protects the scalp from sun damage, and aids in regulating body temperature. Hair strands consist of three distinct layers: the cuticle, cortex, and medulla [1]. The cuticle, the outermost layer, is composed of overlapping, transparent dead cells that act as a protective barrier [2,3]. The cortex, the thickest middle layer, determines the strength, elasticity, and texture of hair. It contains melanin, the pigment responsible for hair color [4]. The medulla, the innermost layer, is a less densely packed region with varying structures depending on hair type [2]. Hair strands are primarily composed of proteins (65–95 wt%), with water (up to 32 wt%) being another major component. Other constituents include pigments (melanin), lipids, minerals, and trace elements [5]. Keratin, a fibrous protein rich in cysteine, is the main structural protein in hair and is responsible for its strength and shape [6]. Keratin itself is composed of various amino acids, including cysteine, methionine, leucine, serine, glycine, arginine, and threonine [6].

Hair is susceptible to both physical and chemical damage. Physical damage often arises from routine practices like combing, while chemical damage typically arises from treatments like perming and dyeing [7]. Chemical hair dyes can be categorized into permanent, semi-permanent, temporary hair dyes, and bleach based on their duration and type of chemical process [8]. Permanent hair dyes provide the longest-lasting color. These dyes use a combination of dye precursors (such as *p*-phenylenediamine, PPD) and an oxidizing agent (such as hydrogen peroxide, H_2_O_2_) [8]. The oxidizing agent opens the hair cuticle, allowing the dye to penetrate into the cortex and medulla. Semi-permanent hair dyes don't use oxidizers; instead, they contain pre-formed color molecules that attach to the outer layer of the hair shaft [8]. So, the color lasts for a period of time, but will fade slightly with each wash. Temporary hair dyes are dyes that are only absorbed into the hair cuticle or simply adhere the colorant to the surface of the hair. These dyes do not have a chemical reaction with the hair and is removed after just a few washes. Bleach uses high concentrations of hydrogen peroxide and sometimes ammonium persulfate ((NH_4_)_2_S_2_O_8_) to remove natural pigment from hair, making it lighter [9]. Bleach reacts chemically with hair, breaking down the melanin in hair fiber.

Hair dye can damage hair through various mechanisms, primarily due to the chemicals involved in the dyeing process. Types of hair damage include structural damage, protein loss, moisture loss, chemical burns, scalp irritation, and oxidative stress [10]. The extent and type of damage depend on the type of dye used (permanent, semi-permanent, etc.), the frequency of dyeing, and the condition of the hair prior to dyeing. This study aimed to quantify the extent of hair damage caused by repeated dyeing in human virgin hair. Hair was dyed and then re-dyed up to 10 times to assess changes. Mechanical and chemical changes due to dyeing were evaluated using various techniques, including Fourier transform infrared spectroscopy (FT-IR), atomic force microscopy (AFM), and tensile strength measurements as a function of the number of dyes. The observed changes in mechanical properties were then linked to the chemical alterations. This study effectively quantified the degree of hair damage as the number of dye cycles increased.

## 2 Material and methods

### 2.1 Hair samples and dyes

The hair sample used in this work were purchased from a company (Beaulax Co., Ltd, Saitama-shi, Japan). These samples were sourced from a single individual and were specially prepared for the evaluation of dyeing and permanent agents. The specifications of the hair samples are as follows: human black hair (item number: BS-PG, Chinese, female, 20s ∼ 30s), length 60 cm, weight 100 g, with hair cuticle arranged in one direction. All the samples were cleaned using a 1 % sodium dodecyl sulfate solution and sonicated for 30 s [11]. The hair sample was then dyed with a commercial product (Excellence Bleach Supreme (74WO0A MFG09082022), L'Oreal, Clichy, FR, USA). The dyeing agent consisted of bleach powder, oil oxidizer, and lightning cream. The dyeing process involved mixing the bleach powder and oil oxidizer for 30 s, followed by the lightning cream for another 30 s to obtain the final mixture. All hair samples were treated with this final solution for 30 min. After washing with water and drying, the hair samples were air-dried at room temperature. Each hair sample, before and after dyeing, was observed under an optical microscope (Objective LD EC Epiplan-Neofluar 50 × /0,55 DIC, ZEISS, Oberkochen Germany) at 50 × magnification. The hair was affixed to a slide glass using carbon tape, and optical images were captured. The total number of hairs used in the experiment was approximately 180. Of these, around 150 hairs were dyed for the first time, and then the characteristics of 20–30 of them were measured. The remaining about 120 hairs were subsequently dyed a second and third time, with 20–30 of these being characterized each time. This process continued, with hair being dyed and characterized up to 10 times.

### 2.2 Attenuated total Reflectance (ART) FT-IR spectroscopy

ATR-FTIR spectra were collected using a Cary 660 FTIR spectrometer (Agilent Technologies, CA, USA). For each spectrum, a single strand of hair was placed on a Diamond MIRacle ATR crystal [12,13]. The press mounted on the ATR was then lowered for analysis. The ATR-FTIR spectra were acquired in the range of 4000-400 cm^−1^ with 256 scans averaged at a resolution of 4 cm^−1^ and a scan speed of 5 kHz. The analysis focused on identifying changes in functional groups, particularly those associated with proteins (e.g., amide bonds).

### 2.3 Atomic force microscopy

The surface topography of the hair fibers was imaged using an AFM (Nano N8 Neos, Bruker®, Germany) in contact mode. Hair fibers were scanned over different areas of 30 × 30 μm^2^, 20 × 20 μm^2^, and 15 × 15 μm^2^ at a resolution of 512 × 512 pixels and a scan rate of 0.4 lines/s [14]. Surface roughness and cuticle step height were analyzed using an image analysis program (Scanning Probe Image Processor; Image Metrology, Denmark). Force–distance curve (FD curve) measurements were employed to determine the mechanical properties of the hair fibers. The FD curve was obtained with a load force of less than 10 nN and a loading velocity of less than 1 μm/s. The detailed information of the contact mode probe (ContGD, Budget Sensors Inc., Sofia, Bulgaria) used is as follows: resonance frequency of 13 kHz (±4 kHz), force constant of 0.2 N/m (0.07–0.4 N/m), cantilever length of 450 μm (±10 μm), cantilever width of 50 μm (±5 μm), cantilever thickness of 2 μm (±1 μm), tip height of 17 μm (±2 μm), and tip radius of <10 nm.

### 2.4 Stress–strain curve measurement

The middle section of each hair fiber was cut to a specific length and secured at both ends with metal crimps. The sample was then mounted on an equipment (Fibra.one, DIA-STRON, East Anton, UK), and a tensile strength test was conducted by applying a gauge force of 2 gf at a rate of 100 mm/min until the fracture point [15]. The stress was calculated through the ***σ* = *F*/*A*** relational expression, where ***σ*** denotes tensile stress applied to the hair fiber, ***F*** is the force applied to the hair, and ***A*** is the cross-sectional area of the hair sample.

### 2.5 Statistical analysis

All data were statistically analyzed and expressed as mean ± standard error of the mean (SEM). Analyses were performed using Student's t-test. Statistical significance was set at p < 0.05. The notation for significance levels was *p ≤ 0.05, **p ≤ 0.01, ***p ≤ 0.001, and ****p ≤ 0.0001. Microsoft Excel (version 2010) was used for the statistical analysis.

## 3 Results and discussion

### 3.1 Effects of dyeing on topography of hair fiber

**Fig. 1:**
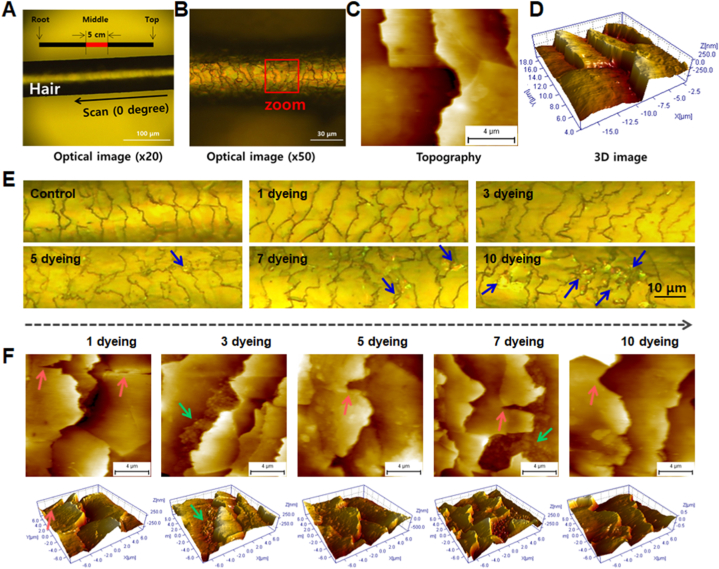
Optical image showing (A) a section of hair used in the experiment and (B) its magnified image. Representative (C) two-dimensional and (D) three-dimensional AFM images of control hair fibers. (E) Optical image of hair fibers dyed up to 10 times ( × 50). (F) Two-dimensional and three-dimensional AFM images of hair fibers dyed up to 10 times. Cuticle debris was occasionally observed, as indicated by green arrows. Morphological damage and cuticular debris are indicated by pink and green arrows, respectively.

Light microscopy and AFM were employed to assess the topography of control and dyed hair fibers. As shown in the optical images (Fig. 1A and B), a 5 cm section was obtained from the midsection of each hair strand for AFM analysis. During AFM imaging, the probe scanned the hair surface vertically, following the alignment of the cuticle scales. This analysis yielded topographic and three-dimensional (3D) images for further evaluation (Fig. 1C and D). The AFM image of the control hair fiber clearly revealed the characteristic "stepped" arrangement of the cuticular scales, indicative of a healthy and well-organized structure. Each cuticle scale was well-defined, and the overall surface appeared smooth and regular. Notably, no foreign material was observed between the cuticles.

To investigate the effects of repeated dyeing cycles, hair fibers were imaged after undergoing 3, 5, 7, and 10 dyeing treatments, compared to the control. Optical images of hair fibers showed changes in cuticle shape due to dyeing (Fig. 1E). As the number of dyeing cycles increased, the cuticle boundaries became ragged and debris (blue arrow) was observed between the cuticles. The microscopic morphological differences between control and dyed hair fibers were further examined with AFM images. The dyed hair fibers exhibited wrinkled or ruptured edges on the cuticle scales, as indicated by the pink arrows in Fig. 1F. The layered structure of the cuticle appeared disrupted, resulting in an irregular and bumpy surface texture. Furthermore, residual endocuticles (innermost layer) and cuticle debris were occasionally detected, highlighted by the green arrows in Fig. 1F. These observations suggest damage to the cuticle caused by the dyeing process. Surface imaging was performed on 20–30 hair fibers from each group of control and dyed hair.

### 3.2 Changes in cuticle structure and surface roughness

**Fig. 2:**
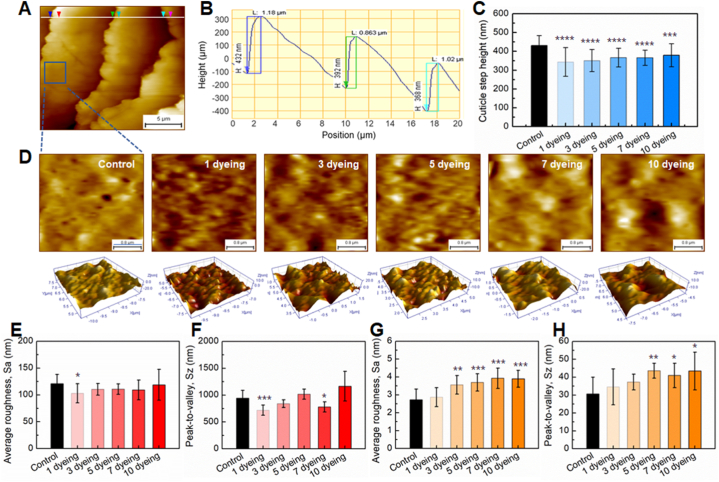
(A) AFM image displaying contour lines for cuticle step height analysis. (B) Line profile extracted from Fig. 2A. (C) Cuticle step height as a function of the number of dyeing processes. (D) 2D and 3D cuticle surface AFM images scanned in an area of 3 × 3 μm^2^ in both control and dyed hair fibers. Cuticle surface roughness parameters (E) Sa and (F) Sz determined from an AFM image of 15 × 15 μm^2^. The roughness parameters (G) Sa and (H) Sz determined from an AFM image of 3 × 3 μm^2^.

To quantify the effect of dyeing on hair fiber structure, the cuticle step height was determined from the AFM images. A contour line drawn across the cuticles in the 2D AFM image generated a line profile (Fig. 2A) used to determine the height of each cuticle (Fig. 2B). The average cuticle step height of control hair fibers was approximately 430.2 ± 52.7 nm (Fig. 2C). Hair fibers dyed once had an average step height of 343.3 ± 75.9 nm, which was 20 % lower than the control. The cuticle step height gradually increased as the number of dyeing increased; however, all the dyed hair samples had a lower step height than the control.

Surface roughness refers to the irregularity of a surface, indicating its lack of smoothness, and is quantified by measuring the average height and depth variations across the surface. In the evaluation of hair surface alterations resulting from dyeing, surface roughness was assessed using AFM images scanned at 15 × 15 μm^2^ and 3 × 3 μm^2^, respectively. A 15 × 15 μm^2^ area typically encompasses three to four cuticles, thereby capturing information about both the cuticle surface and the intercuticular steps (Fig. 2A). Conversely, a smaller area of 3 × 3 μm^2^ provides information solely about the cuticle surface (Fig. 2D). Two parameters, Sa and Sz, were analyzed to characterize surface roughness. Sa represents the dispersion parameter, calculated as the mean of the absolute deviations of surface features from the mean plane within the sampled area [16]. Sz, on the contrary, measures the maximum peak-to-valley height of the profile, and is particularly sensitive to extreme surface irregularities [16]. Sa, assessed over a large area of 15 × 15 μm^2^, exhibited a slight decrease in dyed hair and displayed no correlation with the number of dyeing (Fig. 2E). Interestingly, the changes in Sa mirrored those observed in cuticle step height (Fig. 2C), suggesting that cuticle step height significantly contributes to surface roughness over larger areas. Similarly, Sz measured over a large area demonstrated analogous changes to Sa (Fig. 2F). Conversely, Sa values measured over a smaller area of 3 × 3 μm^2^ exhibited distinct behavior compared to those assessed over a larger area (Fig. 2G). Sa values increased with dyeing, with a statistically significant increase observed after three or more dyeing cycles. Additionally, Sz values of dyed hair were higher compared to those of the control. These findings indicate that although macroscopic damage to the hair surface due to repeated dyeing may not be readily apparent, it is directly proportional to the number of dyeing cycles.

### 3.3 Changes in mechanical property of hair fiber

**Fig. 3:**
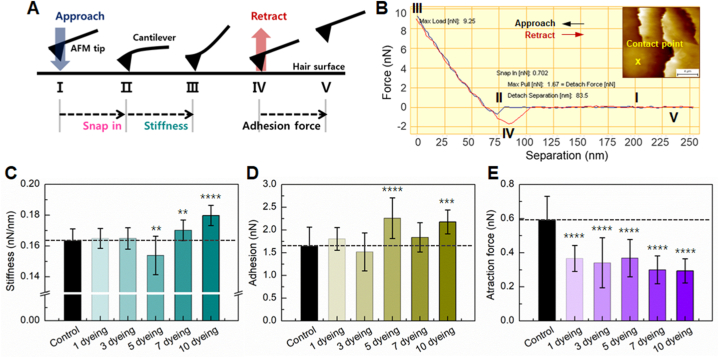
(A) An illustration of the five steps involved in measuring a force-distance curve (FD-curve) using an AFM. (B) A representative FD-curve obtained from a control hair fiber. Changes in (C) stiffness, (D) adhesion force, and (E) attraction force as a function of the number of dyeing processes.

The mechanical properties of hair fibers, including stiffness, adhesion, and attraction force, were determined by measuring force-distance curves (FD curves) using AFM. The FD curve measurement process can be described in five steps (Fig. 3A and B) [17]. Initially, the AFM probe was positioned by selecting a measurement location on the sample, as shown in the inset of Fig. 3B, and then brought close to the tip of the sample (Step I). As the distance between the cantilever and the sample decreased, the attractive force between the tip and the sample caused the cantilever to bend toward the sample surface, momentarily contacting it (Step II, snap-in). Further extension of the probe toward the sample surface to a predetermined setpoint resulted in increased cantilever bending due to the escalating repulsive force (Step III). The stiffness of the sample was indirectly determined from the relationship between the load on the cantilever in this region and the distance between the tip and the sample. Upon reaching the set point, the tip was retracted from the sample surface. Below a certain force threshold, the cantilever bent toward the sample surface due to attractive adhesion (Step IV). Pull-off occurred as the cantilever separated from the surface during further retraction (Step V). The attraction force is derived from the interaction force between the AFM tip and hair surface during snap-in (Step II), while the force separating the AFM tip from the hair surface during retraction in Step IV corresponds to the adhesion force.

Fig. 3C shows the stiffness of hair fibers. Control hair fibers exhibited an approximate stiffness of 0.163 ± 0.007 nN/nm, a value that remained consistent until after three dyeing cycles. Stiffness increased with an increasing number of dyeing cycles beyond seven. Relative to control hair, stiffness increased by 4 % for hair dyed seven times and by 10 % for hair dyed ten times. Notably, stiffness was visibly reduced in hair dyed five times, although the margin of error was large, making it unlikely to be significantly different from control hair. Adhesion did not significantly change with one or three dye treatments but increased with five or more treatments compared to the control (Fig. 3D). Adhesion did not rise proportionally with an increasing number of dyeing cycles. Conversely, dyeing diminished the attraction between the hair surface and the AFM tip (Fig. 3E). In control hair, attraction measured 0.591 ± 0.140 nN, reducing to 0.367 ± 0.076 nN after treatment with one dye. The reduction observed in hair dyed three to five times was comparable to that of hair dyed once (38%–43 % reduction), whereas the reduction in hair dyed seven to ten times was greater (50%–51 % reduction). Dyeing resulted in increased stiffness and adhesion of hair fibers but decreased attraction force. Particularly, significant changes in the mechanical properties of hair fibers occurred when the number of dyeing cycles exceeded 3–5 times.

### 3.4 Changes in tensile properties of hair fiber due to dyeing

**Fig. 4:**
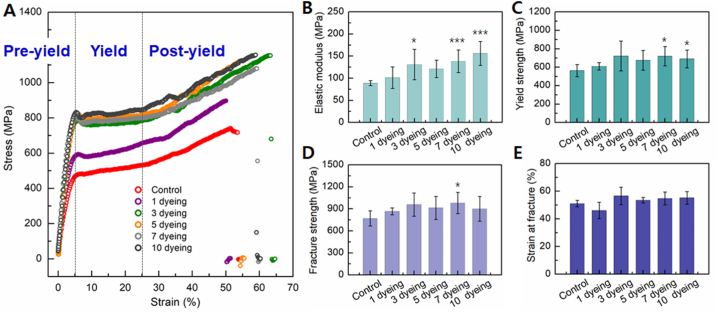
Stress-strain curves for control and dyed hair fibers. Changes in mechanical properties such as (B) elastic modulus, (C) yield strength, and (D) fracture strength presented as a function of the number of dyeing sessions.

The tensile response of human hair serves as a critical measure of its strength and fracture resistance. To gauge the tensile strength of hair fibers, a single strand of hair was clamped before and after dyeing, and tension was applied at both ends. Fig. 4A illustrates the stress-strain curves for both control and dyed hair. The stress–strain curve of human hair typically exhibits three discernible regions: pre-yield, yield, and post-yield [18]. In the pre-yield region, also referred to as the Hookean region, stress and strain display a proportional relationship. Here, α-keratin responds uniformly to stretching stress, with the resistance offered by hydrogen bonds stabilizing the α-helix of the keratin [19]. The slope of the stress-strain curve in the pre-yield region yields the elastic modulus, while the highest stress in this region signifies the yield stress. The yield region marks the transition from α-helices to β-sheet keratin [19]. During this phase, the α-helix structure unfolds without resistance, resulting in minimal change in stress with strain. Conversely, in the post-yield region, β-sheet keratin responds to strain, causing stress to rise again until the hair fiber ultimately fractures [20]. The strain and stress at the point of fiber fracture define the fracture strain and fracture stress, respectively.

The elastic modulus of hair fibers increased with dyeing and showed a proportional relationship with the number of dyeing, except for the seventh dyeing cycle (Fig. 4B). Hair dyed ten times exhibited a 190 % increase in elastic modulus compared to control hair. Both yield strength and fracture strength increased by approximately 40–70 % through dyeing, albeit without a clear correlation with the number of dyeing (Fig. 4C and D). Control hair typically fractured at a strain of around 47 %, while dyed hair fractured at strains exceeding 50 % (Fig. 4E). These findings affirm that dyeing enhances the strength and deformability of hair fibers, with hair deformation being influenced by the number of dyeing cycles. Notably, hair strength increased significantly after just one dyeing cycle, with subsequent changes being less pronounced with an increased number of dyeing cycles.

### 3.5 Changes in chemical structure of hair fiber by dyeing

**Fig. 5:**
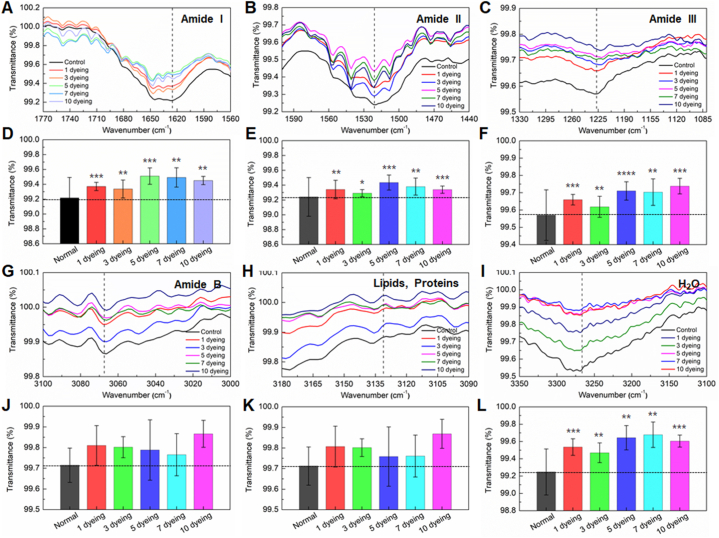
The central frequencies of the (A) amide I, (B) amide II, (C) amide III, (G) amide B, (H) lipid, and (I) water bands in the FT-IR spectrum, along with the intensity of the corresponding bands (D-F, J-L) as a function of the number of dyeing sessions.

Changes in the chemical structure of hair fibers induced by dyeing were investigated using FT-IR microscopy in the range of 4000-400 cm^−1^. The spectra of both control and dyed hairs exhibited characteristic features of keratin protein (Fig. S1). Protein structure is formed through the condensation of amino acids to create peptide bonds [21]. The most prominent peaks appearing at 1600-1690 cm^−1^ and 1480-1575 cm^−1^ were assigned to amide I and amide II, respectively (Fig. 5A and B) [22,23]. The peak observed at 1229-1301 cm^−1^ corresponds to amide III (Fig. 5C) [3]. Amide I represent the strongest absorption band of the protein and is primarily attributed to the stretching vibrations of C=O coupled with the in-plane bending of N-H (70–85 %) and stretching of C-N bonds (10–20 %), which are influenced by the secondary structure (α-helix, β-sheet, etc.) [23]. The transmittance of amide I was increased by dyeing, as depicted in Fig. 5D. Compared to control hair, the transmittance of amide I increased by 16–20 % in hairs dyed 1–3 times, with a further increasing of 30–38 % in hairs dyed 5–10 times. Amide II arises from C-N stretching vibrations coupled with N-H bending [23]. Its transmittance serves as a good indicator of protein folding, reflecting the extent of hydrogen (H) exchanged for deuterium (D) in H-D exchange experiments [24]. Amide III band is highly sensitive to protein secondary structures and remains unaffected by water absorption [25]. The amide III primarily corresponds to CN stretching, NH bending vibrations, CC stretching, and CH bending [26]. These two bands displayed a similar dependence on dyeing, as evidenced by Fig. 5E and F. The transmittance of both amides I and II slightly increased with 1–3 dyeing cycles but significantly diminished with 5–10 dyeing cycles.

The doublet observed at 3066 cm^−1^ was attributed to amide B, associated with the overtones of amide II or a combination of amide I and amide II (Fig. 5G) [21,22]. The weak band observed at 3136–3134 cm^−1^ and the broad band at 3268-3286 cm^−1^ correspond to lipids and water, respectively (Fig. 5H and I). Both the amide B and lipids bands exhibited a similar trend with dyeing (Fig. 5J and K) [27]. The transmittance of both bands increased with dyeing, with the most pronounced increasing observed after 10 dyeing cycles. Dyeing decreased the water content in the hair; notably, the water content of hair dyed more than five times was only 52 % less than that of control hair (Fig. 5L).

**Fig. 6:**
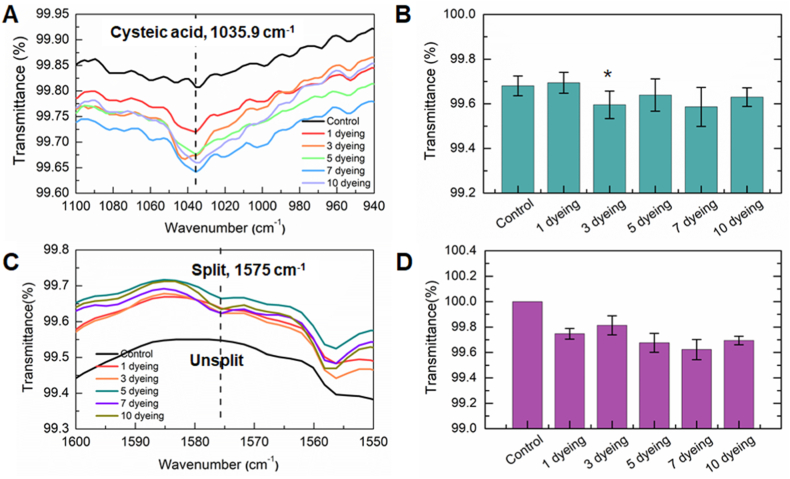
The central frequencies of (A) cysteic acid and (B) the intensity of the band as a function of the number of dyeing sessions. (C) The newly appeared band at 1575 cm^−1^ due to dyeing and (D) the intensity of that band.

As the number of dyeing cycles increased, the transmittance of most FT-IR bands increased, except for the bands at 1035.9 and 1575 cm^−1^, which decreased. The very weak band observed at 1035.9 cm^−1^ in the spectrum of control hair corresponds to the symmetric stretching vibration of the S=O group due to cysteic acid [28] (Fig. 6A). After dyeing, the transmittance and broadening of the cysteic acid band decreased. The transmittance notably decreased after three dyeing cycles, with no further change observed with additional dyeings (Fig. 6B). Cysteic acid is formed by the oxidation of disulfide groups (S-S) during dyeing. Although not clearly observed experimentally (Fig. S1), the S-S band at 508–516 cm^−1^ is damaged by chemical dyeing, leading to conformational changes [29]. A new band, absent in control hair, appeared at 1575 cm^−1^ after dyeing (Fig. 6C). This band, observed in split hairs, was assigned to the N-H scissoring vibration of NH_2_, occurring as the peptide bond breaks [22]. This band emerged after just one dyeing cycle. However, no statistically significant changes were observed in the transmittance or position of the bands with an increasing number of dyeing cycles (Fig. 6D).

## 4 Discussion and conclusion

Chemical hair dyes alter the properties of hair fibers compared to their untreated, "virgin" state. These changes can be categorized into mechanical properties (strength and elasticity), chemical composition, wetting behavior, and moisture retention. In particular, permanent dyeing results in varying degrees of impairment to hair fibers at molecular and cellular levels, affecting the cell membrane complex, melanin granules, cuticle, and cortex [4]. These alterations lead to structural changes, disruption of chemical composition, and modification of physical properties in hair fibers. Extensive research has been conducted using scanning electron microscopy (SEM) techniques and AFM to examine the morphological properties of hair fibers. These studies have explored how hair structure changes based on factors like race, age, and various treatments [[30], [31], [32]].

In this study, we evaluated hair fiber impairment due to successive permanent dyeing using multiple parameters, including morphological changes and cuticle layer surface roughness, mechanical strength analyzed by AFM, stress-strain curves, adhesion and attraction forces, and alterations in chemical compositions determined by FT-IR. The morphological changes induced by dyeing are apparent in our findings. The cuticle exhibits wrinkling or rupture, alongside structural breakdown such as reduced cuticle step height. Moreover, dyeing roughens the cuticle surface, with the degree of roughness correlating with the number of dye applications. Mechanical property alterations due to dyeing are also discernible. Hair fiber stiffness, measured via AFM under compressive force, increases with dyeing, as does the elastic modulus derived from tensile stress. Post-dyeing, adhesion force on hair fibers elevates while attraction force diminishes.

These mechanical changes are associated with water loss, hydrophobic alterations, and disruption of chemical bonds in hair fiber proteins due to dyeing. Hair fibers typically contain around 10 % water, its content fluctuating based on external humidity [33]. Water permeates the outer cuticle layer, lifting it and allowing water to penetrate deeper into the hair structure. Thus, increased water absorption leads to heightened cuticle step height. As per O'Connor, soaking hair in water for 5 min increases cuticle step height from 460 to 675 nm [34]. In our study, hair fiber water content decreased by 40 % after initial dyeing, further decreasing with successive dyeing (Fig. 5L). Therefore, the observed reduction in cuticle step height of dyed hair (Fig. 3C) can be attributed to water loss due to dyeing.

Water content also influences alterations in hair fiber mechanical properties. Immersion in water typically reduces hair fiber tensile strength and elastic modulus [35], indicating softer hair due to water absorption. Firstly, water can alter chemical interactions, primarily affecting the elastic modulus, which is mainly due to α-helix hydrogen bond resistance, with Coulomb interaction bonds contributing to some extent [36]. Increased water content may break some Coulomb interaction bonds as water displaces binding sites, resulting in lower wet elastic modulus. Secondly, hair diameter swells in water, potentially contributing to softness. Human hair diameter expands by 14–16 % in water [1]. In our study, various mechanical parameters of hair, including stiffness, elastic modulus, yield strength, breaking strength, and strain at break, increased with dyeing time. Thus, these mechanical property changes may be attributed to molecular bonding and geometrical alterations due to reduced hair water content.

A notable finding of this study is that significant structural, mechanical, and chemical damage to hair occurs after just one dyeing, with the extent of damage not directly proportional to the number of dye applications. While stiffness, adhesion, amides I, II, and III, and water changes show no significant difference with 1–3 dyeing cycles, damage intensifies significantly after five or more applications. However, nanoscale damage such as cuticle surface roughness is proportional to the number of dye applications.

With the beauty and personal care industry booming, hair-related products such as hair dyes and perms are becoming increasingly popular among all age groups. Consequently, people are coloring their hair more frequently and using a wider variety of chemical hair dyes. Meanwhile, growing concerns about hair damage have led to the research and development of new products designed to reduce hair damage, such as ammonia-free hair dyes and organic hair dyes [37,38]. Although the degree of hair damage caused by chemical products has been reduced compared to the past, the issue of accumulated damage from repeated dyeing has not been fully addressed. In this study, we measured the mechanical and chemical changes in hair due to dyeing using AFM and FT-IR, demonstrating that it is possible to evaluate the accumulation of fine hair damage from repeated dyeing. Contrary to expectations, the degree of hair damage was not directly proportional to the number of dyeing cycles, with the first and fifth dyeing causing particularly severe damage. The purpose of this study was to quantitatively analyze the degree of hair damage caused by repeated dyeing. Therefore, the hair from the same individual was repeatedly dyed and changes were observed. To generalize the quantification of damage caused by repeated dyeing identified in this study, we plan to conduct future studies on hair from diverse populations (varying in age, gender, race, etc.) using the same methodology.

## Data availability

The datasets used and/or analyzed in the current study are available from the corresponding author upon reasonable request.

## CRediT authorship contribution statement

**Sangwoo Kwon:** Writing – original draft, Methodology, Data curation. **Seoyoon Lee:** Methodology, Data curation. **Jihui Jang:** Methodology, Data curation. **Jun Bae Lee:** Writing – review & editing, Writing – original draft, Conceptualization. **Kyung Sook Kim:** Writing – review & editing, Writing – original draft, Supervision, Conceptualization.

## Declaration of competing interest

The authors declare that they have no known competing financial interests or personal relationships that could have appeared to influence the work reported in this paper.

## References

[1] Robbins C.R. (2012).

[2] Yang F.C., Zhang Y., Rheinstädter M.C. (2014). The structure of people's hair. PeerJ.

[3] Swift J.A., Smith J.R. (2001). Microscopical investigations on the epicuticle of mammalian keratin fibres. J. Microsc..

[4] He Y., Cao Y., Nie B., Wang J. (2023). Mechanisms of impairment in hair and scalp induced by hair dyeing and perming and potential interventions. Front. Med..

[5] Cloete E., Khumalo N.P., Van Wyk J.C., Ngoepe M.N. (2019). Systems approach to human hair fibers: interdependence between physical, mechanical, biochemical and geometric properties of natural healthy hair. Front. Physiol..

[6] Shimomura Y., Ito M. (2005). Human hair keratin-associated proteins. J. Invest. Dermatol. Symp. Proc..

[7] Lee, Kim Y.D., Pi L.Q., Lee S.Y., Hong H., Lee W.S. (2014). Comparison of hair shaft damage after chemical treatment in Asian, White European, and African hair. Int. J. Dermatol..

[8] Guerra-Tapia A., Gonzalez-Guerra E. (2014). Hair cosmetics: dyes. Actas Dermosifiliogr.

[9] Nagai A., Komoriya H., Bunai Y., Yamada S., Jiang X.Y., Ohya I. (1991). Effect of hair dyes and bleach on the hair protein patterns as revealed by isoelectric focusing. Electrophoresis.

[10] Camargo F.B. Jr., Minami M.M., Rossan M.R., Magalhães W.V., Porto Ferreira V.T., Maia Campos P.M.B.G. (2022). Prevention of chemically induced hair damage by means of treatment based on proteins and polysaccharides. J. Cosmet. Dermatol..

[11] Jeong K.H., Kim K.S., Lee G.J., Choi S.J., Jeong T.J., Shin M.K., Park H.K., Sim W.Y., Lee M.H. (2011). Investigation of aging effects in human hair using atomic force microscopy. Skin Res. Technol..

[12] Kim K.S., Ks H.K. (2013). Analysis of aging effects on chemical property of human hair by Fourier transform infrared spectroscopy. Skin Res. Technol..

[13] Kim K.S., Shin M.K., Park H.K. (2012). Effects of ultraviolet B radiation on physicochemical properties of human hair shaft. Microsc. Res. Tech..

[14] Kim K.S., Shin M.K., Ahn J.J., Haw C.R., Park H.K. (2011). Investigation of hair shaft in seborrheic dermatitis using atomic force microscopy. Skin Res. Technol..

[15] Robinson M.S., Rigby B.J. (1985). Thiol differences along keratin fibers: stress/strain and stress-relaxation behavior as a function of temperature and extension. Textil. Res. J..

[16] Park K.H., Kim H.J., Oh B., Lee E., Ha J. (2018). Assessment of hair surface roughness using quantitative image analysis. Skin Res. Technol..

[17] Kwon S., Yang W., Moon D., Kim Ks K.S. (2020). Comparison of cancer cell elasticity by cell type. J. Cancer.

[18] Indira P.S., Bharat B. (2008). Effect of ethnicity and treatments on in situ tensile response and morphological changes of human hair characterized by atomic force microscopy. Acta Mater..

[19] Yang Y., Wang Y., Bin W., Marc M.A. (2017). Structure and mechanical behavior of human hair. Mater. Sci. Eng. C.

[20] Kreplak L., Doucet J., Dumas P., Briki F. (2004). New aspects of the α-helix to β-sheet transition in stretched hard α-keratin fibers. Biophys. J..

[21] Cruz C.F., Azoia N.G., Matamá T., Cavaco-Paulo A. (2017). Peptide-protein interactions within human hair keratins. Int. J. Biol. Macromol..

[22] Pienpinijtham P., Thammacharoen C., Naranitad S., Ekgasit S. (2018). Analysis of cosmetic residues on a single human hair by ATR FT-IR micro spectroscopy. Spectrochim. Acta Mol. Biomol. Spectrosc..

[23] Panayiotou H., Kokot S. (1999). Matching and discrimination of single human-scalp hairs by FT-IRmicro-spectroscopy and chemometrics. Anal. Chim. Acta.

[24] Mathew S.B., Kyle C.D., Ray W., Igor K.L. (2017). Differentiation of hair using ATR FT-IR spectroscopy: a statistical classification of dyed and non-dyed hairs. Forensic Chemistry.

[25] Chiaramaria S., Lisa V., Elisa M., Giovanni B. (2020). FTIR investigation of the secondary structure of type I collagen: new insight into the amide III band. Spectrochim. Acta Mol. Biomol. Spectrosc..

[26] Sang-Ho L., Noemi G.M., Samuel K. (1999). A quantitative anharmonic analysis of the amide A band in-helical poly(L-Alanine). Biopolymers.

[27] Barba C., Oliver M.A., Martí M., Kreuzer M., Coderch L. (2022). Lipid distribution on ethnic hairs by Fourier transform infrared synchrotron spectroscopy. Skin Res. Technol..

[28] Kuzuhara A. (2005). Analysis of structural change in keratin fibers resulting from chemical treatments using Raman spectroscopy. Biopolymers.

[29] Yuuki T., Minori I., Shoji T. (2008). Effects of permanent waving and bleaching treatments on damage of human hair. Transactions of the Materials Research Society of Japan.

[30] Wang N., Barfoot R., Butler M., Durkan C. (2018). Effect of surface treatments on the nanomechanical properties of human hair. ACS Biomater. Sci. Eng..

[31] Man Q., Zhang L., Cho Y. (2021). Efficient hair damage detection using SEM images based on convolutional neural network. Appl. Sci..

[32] Coroaba A., Chiriac A.E., Sacarescu L., Pinteala T., Minea B., Ibanescu S.A., Pertea M., Moraru A., Esanu I., Maier S.S., Chiriac A., Pinteala M. (2020). New insights into human hair: SAXS, SEM, TEM and EDX for Alopecia Areata investigations. PeerJ.

[33] Barba C., Méndez S., Martí M., Parra J.L., Coderch L. (2009). Water content of hair and nails. Thermochim. Acta.

[34] O' Connor S.D., Komisarek K.L., Baldeschwieler J.D. (1995). Atomic force microscopy of human hair cuticles: a microscopic study of environmental effects on hair morphology. J. Invest. Dermatol..

[35] Seshadri I., Bhushan B. (2008). Effect of ethnicity and treatments on in situ tensile response and morphological changes of human hair characterized by atomic force microscopy. Materials Science Acta Materialia.

[36] Feughelman A. (1997).

[37] Draelos Z.D. (2022). A clinical evaluation of a permanent hair dye designed to reduce allergic contact dermatitis and hair damage. J. Cosmet. Dermatol..

[38] Cui H., Xie Q., Hua Z., Cao L., Xiong Z., Tang Y., Yuan Z. (2022). Recent advancements in natural plant colorants used for hair dye applications: a review. Molecules.

