# Supplementary material for: Quantifying the effects of repeated dyeing: Morphological, mechanical, and chemical changes in human hair fibers
Source: Heliyon. 2024 Sep 12;10(18):e37871. doi: 10.1016/j.heliyon.2024.e37871 (PMC11417257; doi:10.1016/j.heliyon.2024.e37871)

Supplementary material

Quantifying the effects of repeated dyeing: morphological, mechanical, and chemical changes in human hair fibers

Sangwoo Kwon[1], Seoyoon Lee[2], Jihui Jang[2], Jun Bae Lee[2†], Kyung Sook Kim[1,3†]

[1]Department of Biomedical Engineering, College of Medicine, Kyung Hee University, Seoul, 02447, Republic of Korea

[2]Department of innovation, Innovation Lab, Cosmax R&I, Gyeonggi-do, Republic of Korea

[3]Department of Biomedical Engineering, Graduate school, Kyung Hee University, Seoul, 02447, Republic of Korea

Fig. S1 (A) Representative FT-IR spectra of human hair fibers. (B) Spectra obtained from control hair and hair dyed 1, 3, 5, 7, and 10 times.

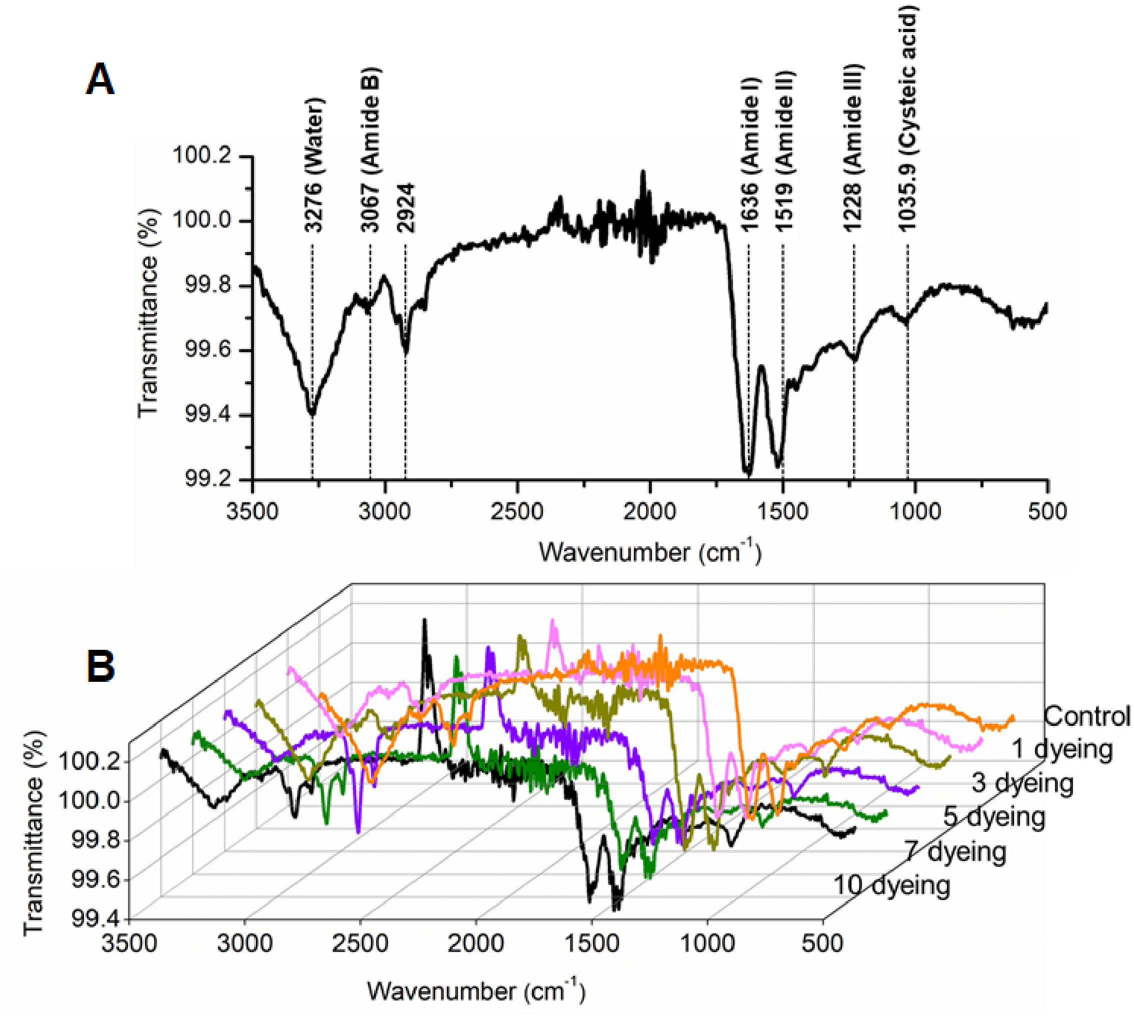

Supplement: Multimedia component 1 [file mmc1.pdf]
